# Lipidomic Analysis of the Protective Effects of Shenling Baizhu San on Non-Alcoholic Fatty Liver Disease in Rats

**DOI:** 10.3390/molecules24213943

**Published:** 2019-10-31

**Authors:** Yuanjun Deng, Maoxing Pan, Huan Nie, Chuiyang Zheng, Kairui Tang, Yupei Zhang, Qinhe Yang

**Affiliations:** School of Traditional Chinese Medicine, Jinan University, Guangzhou 510632, China; jnudeng@foxmail.com (Y.D.);

**Keywords:** non-alcoholic fatty liver disease, lipidomics, sirtuin 1, Shenling Baizhu San, traditional Chinese medicine

## Abstract

Shenling Baizhu San (SLBZS), a famous traditional Chinese medicine, has been demonstrated to exert protective effects against non-alcoholic fatty liver disease (NAFLD), but its exact mechanisms have not been well understood. The aim of this study was to investigate the mechanisms underlying the protective effects of SLBZS in a rat model of NAFLD using lipidomics and to evaluate the role of Sirtuin 1 (SIRT1) in the mechanism of SLBZS against NAFLD. The rat model of NAFLD was induced by high-fat feeding. An ultra-performance liquid chromatography-mass spectrometry (UHPLC-MS)-based untargeted lipidomics approach was applied to analyze hepatic lipid alterations, and the SIRT1-selective inhibitor EX 527 was used to inhibit SIRT expression in the liver. The results of body and biochemical parameters, as well as histological changes, indicated that SLBZS administration exerted protective effects against NAFLD. Lipidomic analysis showed that 30 lipid species were effectively regulated by SLBZS administration in rats fed a high-fat diet. Pathway analysis indicated that glycerophospholipid metabolism and glycerolipid metabolism were potential target pathways closely involved in the mechanism of SLBZS against NAFLD. Moreover, the beneficial effects of SLBZS on hepatic steatosis, some biochemical parameters and hepatic lipid species were partly diminished by SIRT1 inhibition. In conclusion, our results suggested that SLBZS administration could effectively alter some hepatic lipid species in rats fed a high-fat diet, which was mainly associated with the regulation of glycerophospholipid and glycerolipid metabolism. Furthermore, the beneficial effects of SLBZS on hepatic lipid metabolism may be at least partly attributed to SIRT1 activation in the liver.

## 1. Introduction

Non-alcoholic fatty liver disease (NAFLD), the most common chronic liver disease worldwide [1], is characterized by excessive fat accumulation in the liver without other causes, such as significant alcohol consumption or the use of steatogenic medication [2]. NAFLD includes a wide spectrum of liver disorders, ranging from simple steatosis to aggressive lesions, including non-alcoholic steatohepatitis (NASH), liver fibrosis and cirrhosis [3]. The prevalence of NAFLD in the general population is estimated at approximately 25% and is increasing in line with obesity [4]. NAFLD is considered to be closely related to metabolic syndrome, which is characterized by the presence of obesity, hyperlipidemia, hypertension, and elevated fasting blood sugar [5]. Although a growing number of studies have made steady progress in clarifying the pathogenesis of NAFLD, the underlying mechanisms of NAFLD are not yet fully understood, and therapeutic options are still limited [6].

Lipids are a unique group of compounds involved in a large number of structural and biological functions in cells to maintain homeostasis [7,8]. Dysregulation of lipid metabolism has been demonstrated to be closely linked with various common metabolic diseases, such as NAFLD [9], obesity [10], and type 2 diabetes mellitus [11]. Lipidomics, a branch of the field of metabolomics, is a biomedical systems-level analysis aiming to characterize lipid species and investigate the complex biological metabolic networks of lipids in biological systems [12]. In recent years, the emergence of advanced analytical techniques for metabolomics has allowed the high-throughput detection of thousands of intact lipid species in various pathophysiological conditions. Specifically, untargeted lipidomics, which focuses on detecting every lipid species present in a biological sample, is considered to be powerful for studying lipid metabolism and molecular mechanisms [13]. Therefore, lipidomics has made it possible to reveal the dynamic and key roles of lipid metabolism in NAFLD, thereby contributing to our better understanding of the pathogenesis of this disease.

In traditional Chinese medicine (TCM), Shenling Baizhu San (SLBZS), is a famous formula derived from “Tai Ping Hui Min He Ji Ju Fang” (Formulas from the Imperial Pharmacy), which was a clinical book of TCM officially compiled during the Song dynasty about a thousand years ago. Our previous study has demonstrated the protective effects of SLBZS on high-fat diet-induced NAFLD rats [14]. In addition, our other study preliminarily indicated that SLBZS could improve hepatic steatosis and activate Sirtuin 1 (SIRT1) in the liver [15]. SIRT1 is considered as a key metabolic sensor involved in many cellular processes and plays a vital role in regulating hepatic lipid metabolism [16,17]. However, the exact mechanism underlying the protective effect of SLBZS against NAFLD is not fully understood due to the multi-level and multi-target effect of herbal medicines. Moreover, the effect of SLBZS on SIRT1 needs to be further studied. In agreement with the holistic thinking of TCM, many metabolomic technologies have been widely applied to investigate the pharmacological bioactivity and biochemical mechanism of TCM [18]. Recently, there have been accumulating studies investigating the therapeutic mechanism of TCM through lipidomic analysis [19]. Therefore, lipidomics may be helpful in revealing the underlying mechanism of SLBZS against NAFLD.

In the present study, we attempted to investigate the mechanisms underlying the protective effects of SLBZS in a rat model of NAFLD, with a focus on hepatic lipid metabolism, using ultra-performance liquid chromatography-mass spectrometry (UHPLC-MS)-based untargeted lipidomics. Meanwhile, we evaluated the role of SIRT1 in the mechanism of SLBZS against NAFLD. To our knowledge, this study was the first to investigate the protective effects of SLBZS on NAFLD rats through a UHPLC-MS-based untargeted lipidomics approach. Furthermore, we hope our study might provide new insights into the application of lipidomics in the modernization of TCM.

## 2. Results

### 2.1. Effect of SLBZS on the Rat Model of NAFLD Induced by a High-Fat Diet

**Figure 1:**
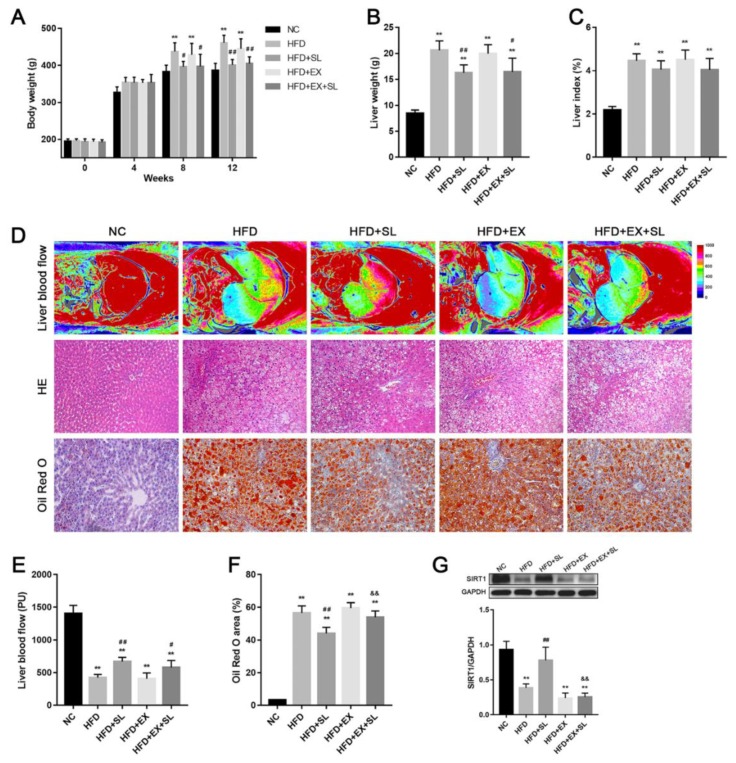
Body characteristics and liver histology in the five groups. (**A**) Body weight. (**B**) Liver weight. (**C**) Liver index. (**D**) Representative images of liver blood flow (upper panel), HE staining (middle panel, ×200 magnification) and Oil Red O staining (bottom panel, ×200 magnification) of the liver. (**E**) Quantification of liver blood flow. (**F**) Quantification of Oil Red O-positive area. (**G**) Western blot analysis of SIRT1 expression in the liver. ** *p* < 0.01, compared with NC group; # *p* < 0.05, ## *p* < 0.01, compared with HFD group; && *p* < 0.01, compared with HFD+SL group.

Body weight changes among groups during the study period are shown in Figure 1A. Before feeding a high-fat diet, there were no significant differences among the five groups for body weight. After feeding a high-fat diet for 8 and 12 weeks, body weight in HFD group was significantly increased compared with that in NC group. However, body weight was significantly decreased in HFD+SL and HFD+EX+SL groups compared with that in HFD group. At the end of the study period, the liver weight and liver index were significantly increased in HFD group compared with those in NC group, while SLBZS administration significantly reduced the liver weight compared with that of HFD group (Figure 1B). The liver index was slightly decreased in HFD+SL and HFD+EX+SL groups compared with that in HFD group, although it did not reach significance (Figure 1C).

To assess the effect of high-fat feeding on liver blood flow, we used a laser perfusion imager to detect the blood flow of the rat liver at the end of the study period. As shown in Figure 1D,E, after feeding a high-fat diet for 12 weeks, the blood flow of the rat liver was significantly decreased compared with that in NC group, whereas SLBZS administration improved liver blood flow compared with that in HFD group. The liver histology of the five groups was examined by HE and Oil Red O staining. As shown in Figure 1B, rat livers of NC group exhibited a normal histological structure without steatosis. In HFD group, typical hepatic steatosis was observed, as evidenced by excessive small lipid droplets inside the cytoplasm and the swelling of hepatocytes. After SLBZS administration, histological changes were markedly improved in HFD+SL group, whereas histological changes in HFD+EX+SL group were slightly improved. As expected, the percentage of Oil Red O area was significantly increased in HFD group compared with that in NC group. In contrast, the percentage of Oil Red O area was significantly lower in HFD+SL group than that in HFD group (Figure 1F). These results indicated the successful establishment of the NAFLD model and the efficacy of SLBZS in mitigating NAFLD.

To further explore the mechanism by which SLBZS protects against NAFLD, we used the SIRT1-selective inhibitor EX 527 to further investigate the effects of SLBZS. As shown in Figure 1G, liver SIRT1 expression was significantly upregulated in HFD+SL group compared with that in HFD group, whereas HFD+EX+SL group showed a decrease in SIRT1 expression similar to HFD and HFD+EX groups. This result indicated that EX 527 effectively abolished SIRT1 activation induced by SLBZS.

**Figure 2:**
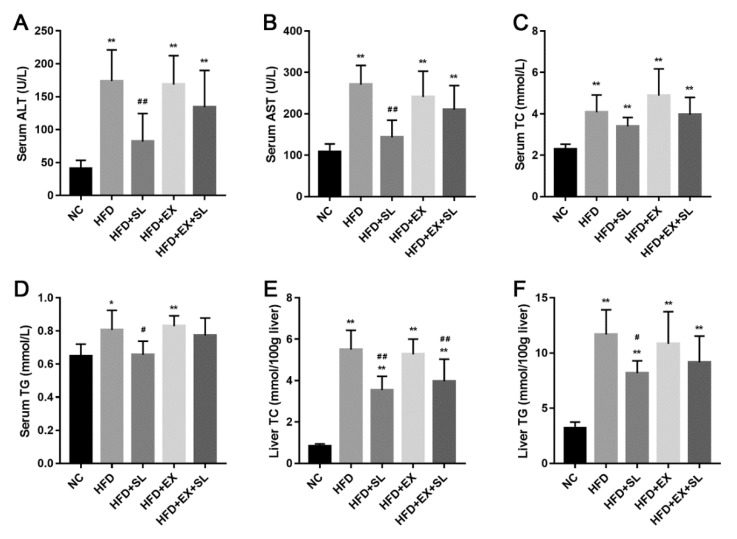
Biochemical parameters of the five groups. (**A**) Serum ALT level. (**B**) Serum AST level. (**C**) Serum TC level. (**D**) Serum TG level. (**E**) Liver TC level. (**F**) Liver TG level. ** *p* < 0.01, compared with NC group; # *p* < 0.05, ## *p* < 0.01, compared with HFD group.

Furthermore, changes in the biochemical parameters in serum and liver were determined. As shown in Figure 2, compared with NC group, the serum levels of ALT, AST, TC and TG were significantly increased in HFD group. Similarly, the liver levels of TC and TG were significantly higher in HFD group than those in NC group. In contrast, SLBZS administration effectively attenuated these abnormalities in biochemical parameters caused by high-fat feeding. However, the protective effect of SLBZS on the biochemical parameters was partly diminished by EX 527, and only the liver TC level was significantly decreased in HFD+EX+SL group when compared with that in HFD group.

### 2.2. Overview of the Lipidomic Analysis of Liver Samples from Different Groups

**Figure 3:**
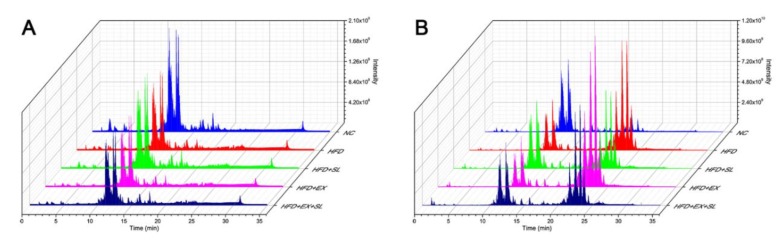
Representative total ion current chromatograms of the five groups in negative ion mode (**A**) and positive ion mode (**B**).

**Figure 4:**
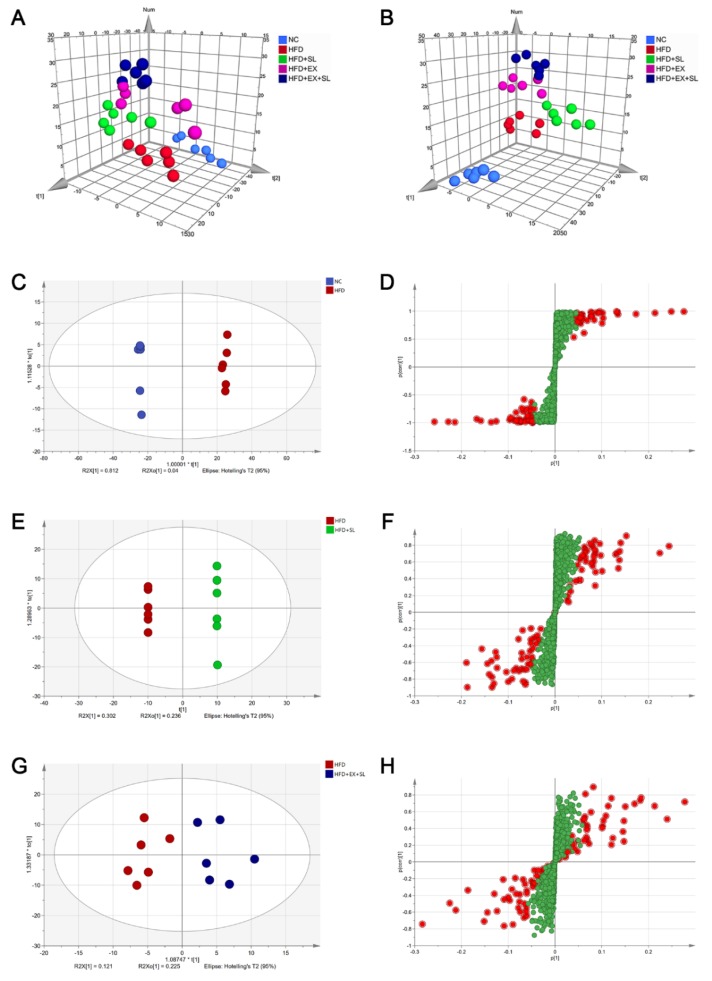
Score plots of the PCA, PLS-DA and OPLS-DA models of the five groups. (**A**) 3D PCA score plot of the five groups [R2X(cum) = 0.761, Q2(cum) = 0.687]. (**B**) 3D PLS-DA score plot of the five groups [R2X(cum) = 0.757, Q2(cum) = 0.357]. (**C**) OPLS-DA score plot between NC group and HFD group [R2X(cum) = 0.852, Q2(cum) = 0.995]. (**D**) S-plot between NC group and HFD group. (**E**) OPLS-DA score plot between HFD group and HFD + SL group [R2X(cum) = 0.88, Q2(cum) = 0.845]. (**F**) S-plot between HFD group and HFD + SL group. (**G**) OPLS-DA score plot between HFD group and HFD + EX + SL group [R2X(cum) = 0.347, Q2(cum) = 0.087]. (**H**) S-plot between HFD group and HFD + EX + SL group. Potential variables in S-plot are marked in red when VIP >1.5.

Representative UPLC-MS total ion current chromatograms of the five groups in negative and positive ion modes are shown in Figure 3. The reliability and reproducibility of the whole analysis were evaluated by the QC samples used during the experiment. The base peak intensity chromatograms of the QC samples showed that the response intensity and retention time of each peak overlapped (Supplementary Figure S2). In addition, the PCA score plot of all samples showed that the QC samples were grouped together and mainly distributed in the middle of all samples (Supplementary Figure S3). These results confirmed the good reliability and reproducibility of our analytical method. Finally, a total of 1055 lipid species belonging to 32 lipid classes were identified in this study (Supplementary Figure S4). To investigate the lipid alterations among different groups, multivariate analysis was performed with SIMCA software. According to the score plots of the PCA (Figure 4A) and PLS-DA (Figure 4B), the NC, HFD and HFD+SL groups were well separated from each other, suggesting the success of modeling and SLBZS administration.

### 2.3. Distinct Changes in the Lipid Species among Groups

Furthermore, OPLS-DA models were established and employed to better identify differential lipid species. The OPLS-DA score plot exhibited a clear distinction between NC group and HFD group (Figure 4C). Likewise, there was a complete separation of HFD group from HFD+SL group (Figure 4E), whereas the difference between HFD group and HFD+EX+SL group was less obvious (Figure 4G). In the S-plots, the red circles denote the important variables with VIP > 1.5 (Figure 4D,F,H). The variables with VIP values >1.5 and *p* values of unpaired Student’s t-test < 0.05 were considered to be significantly changed lipids.

**Figure 5:**
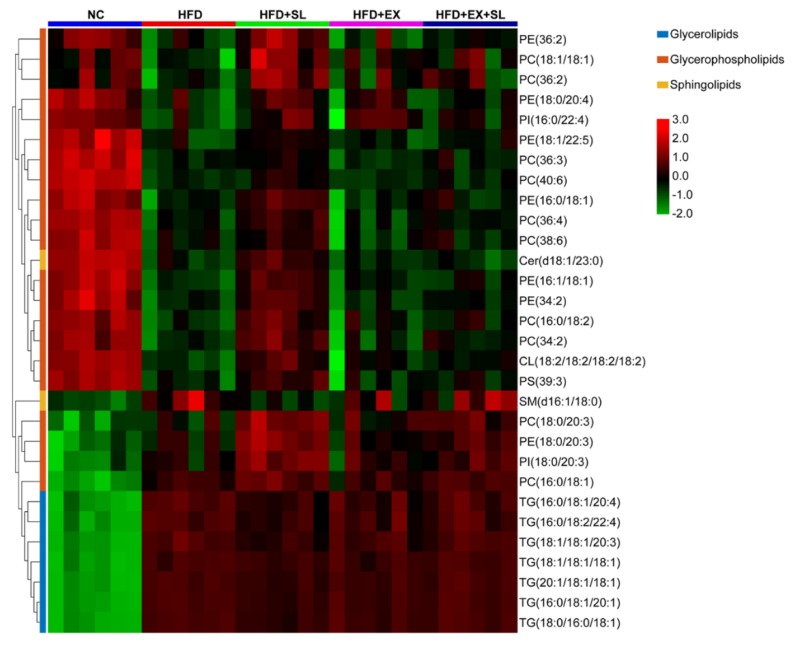
Heatmap of the differential lipid species among the five groups. Red denotes a relative increase, and green denotes a relative decrease.

**Figure 6:**
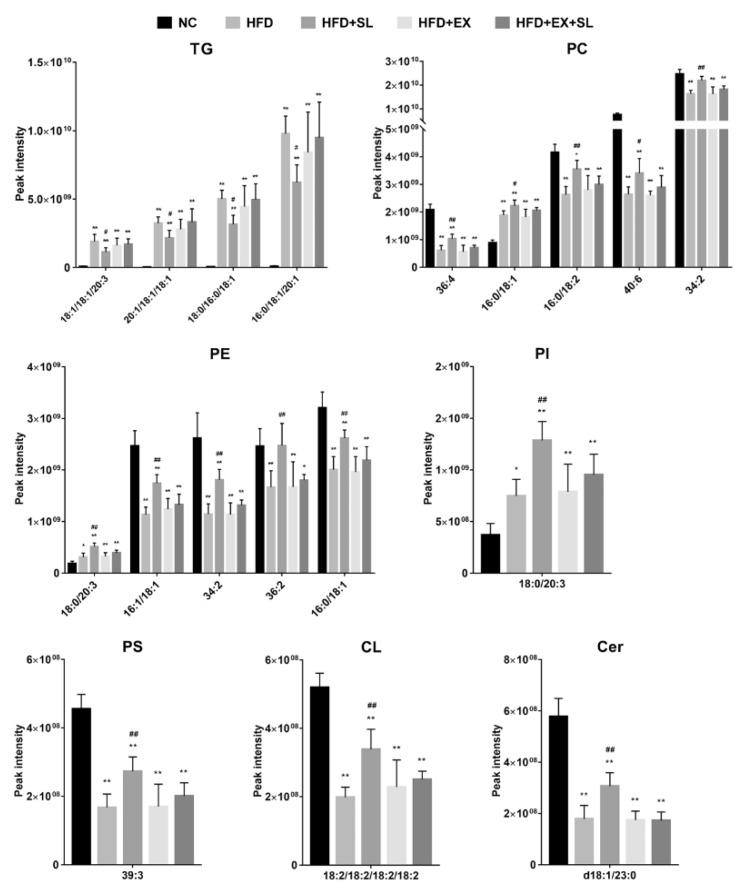
Intensities of representative differential lipid species of the five groups. ** *p* < 0.01, compared with NC group; # *p* < 0.05, ## *p* < 0.01, compared with HFD group.

As shown in Supplementary Figure S5 and Table S2, compared with NC group, 67 lipid species, including 20 triglycerides (TGs), 1 cardiolipin (CL), 1 lysophosphatidylcholine (LPC), 2 lysophosphatidylethanolamines (LPEs), 22 phosphatidylcholines (PCs), 12 phosphatidylethanolamines (PEs), 3 phosphatidylinositols (PIs), 2 phosphatidylserines (PSs), 1 ceramide (Cer) and 3 sphingomyelins (SMs), were significantly changed in HFD group. Compared with HFD group, 47 lipid species and 8 lipid species were significantly changed in HFD+SL group and HFD+EX+SL group, respectively (Supplementary Figure S5, Tables S3 and S4). Among them, 30 lipid species were also significantly changed in HFD group relative to NC group (Supplementary Figure S5). As shown in Figure 5 and Table 1, there were 7 TGs, 1 CL, 10 PCs, 7 PEs, 2 PIs, 1 PS, 1 Cer and 1 SM in the 30 lipid species, which could be classified into three categories, including glycerophospholipids, glycerolipids and sphingolipids. Compared with NC group, the hepatic levels of TG species and SM species were significantly increased in HFD group, whereas the levels of most PC, PE, PI and PS species, as well as the levels of Cer species, were significantly decreased. Most of these lipid species were significantly reversed by SLBZS administration. Interestingly, 4 lipid species, including PC(16:0/18:1), PC(18:0/20:3), PE(18:0/20:3) and PI(18:0/20:3), were significantly increased in HFD group, and SLBZS administration further increased these glycerophospholipids (Table 1, Figure 6). In addition, there were only 3 lipid species, including CL(18:2/18:2/18:2/18:2), PE(16:1/18:1) and PE(36:2), that were significantly increased in HFD+EX+SL group compared with levels in HFD group. The above results indicated that the protective effect of SLBZS on the rat model of NAFLD may be attributed to the regulation of these differential lipids, and SIRT1 inhibition by EX 527 may partly diminish the effect of SLBZS.

### 2.4. Pathway Analysis of Differential Lipid Species

**Figure 7:**
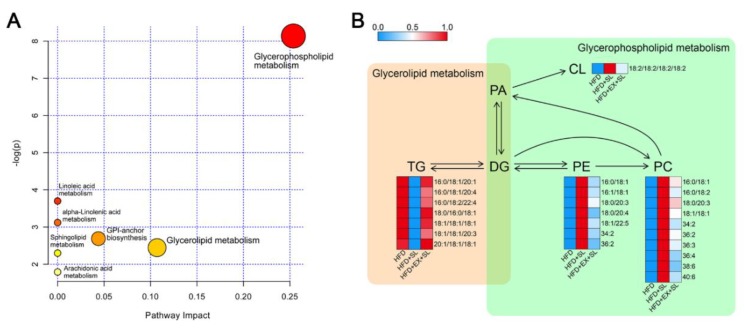
Lipid metabolic pathway analysis of differential lipid species. (**A**) Bubble chart of the pathway analysis by MetaboAnalyst. (**B**) Summary of the main relationships among differential lipid species related to glycerophospholipid and glycerolipid metabolism. Red denotes a relative increase, and blue denotes a relative decrease.

The metabolic pathways relevant to the 30 differential lipid species were determined using the pathway analysis module of MetaboAnalyst 4.0. The pathway analysis results showed that differential lipid species were involved in 7 metabolic pathways, including glycerophospholipid metabolism, glycerolipid metabolism, glycosylphosphatidylinositol (GPI)-anchor biosynthesis, linoleic acid metabolism, alpha-linolenic acid metabolism, sphingolipid metabolism, and arachidonic acid metabolism (Table 2, Figure 7A). Among them, glycerophospholipid metabolism and glycerolipid metabolism were considered as potential target pathways according to the impact values of the metabolic pathways. The relationship between the differential lipid species in these two pathways is summarized in Figure 7B. The metabolic networks of differential lipid species were visualized by the MetScape App in the Cytoscape software platform based on the Kyoto Encyclopedia of Genes and Genomes (KEGG) database (Supplementary Figure S6). These results indicated that both glycerophospholipid and glycerolipid metabolism might play important roles in the progression of NAFLD and the protective effect of SLBZS.

## 3. Discussion

The growing prevalence of NAFLD has brought increasing clinical and economic burdens to many countries [20]. Nevertheless, until now, there has still been a lack of consensus on effective pharmacotherapies for the disease [6]. In recent years, many herbal medicines have been demonstrated to be potential therapeutic agents for the prevention and treatment of NAFLD [21,22]. Our previous studies preliminarily demonstrated that SLBZS could exert a protective effect on NAFLD and activate SIRT1 in the liver [14,15]. However, the exact mechanism remains obscure. Therefore, in this study, we followed our previous studies and further investigated the effect of SLBZS on a rat model of NAFLD. The histologic results showed that rats fed a high-fat diet for 12 weeks developed typical hepatic steatosis. Moreover, high-fat feeding resulted in increased body weight, liver weight and liver index, as well as abnormalities in biochemical parameters. These results suggest that we successfully established a rat model of NAFLD by high-fat feeding, which was in agreement with other studies [23,24]. Consistent with our previous studies, SLBZS administration effectively attenuated hepatic steatosis and reduced body weight and liver weight. Moreover, abnormalities in biochemical parameters caused by high-fat feeding were markedly improved by SLBZS. In addition, we investigated the effect of SLBZS on liver blood flow. As expected, SLBZS markedly improved the decreased liver blood flow induced by high-fat feeding. Together, these results demonstrated the protective effect of SLBZS against NAFLD in rats.

It is well established that SIRT1 plays important roles in hepatic lipid metabolism and the development of NAFLD [17]. To better understand the role of SIRT1 in the effect of SLBZS against NAFLD, we followed up our previous findings and inhibited the expression of SIRT1 in the liver by the SIRT1-selective inhibitor EX 527. As expected, SLBZS administration markedly induced SIRT1 activation in the liver, which was consistent with our previous study [15]. In contrast, EX 527 intervention effectively inhibited the increased expression of SIRT1 induced by SLBZS, and partly diminished the beneficial effects of SLBZS on hepatic steatosis, liver blood flow and biochemical parameters in rats fed a high-fat diet. Recently, there is some evidence that *Panax ginseng*, the major component of SLBZS, may contribute to the effect of SLBZS on SIRT1 activation. Some active ingredients from *Panax ginseng*, such as Ginsenosides Rb1 and Rb2, have been reported to activate SIRT1 expression, and thereby increase fatty acid oxidation, as well as decrease hepatic lipid accumulation [25,26,27]. Therefore, further investigations on the mechanisms of these potential active ingredients from *Panax ginseng* may help to better understand the exact mechanism of SLBZS against NAFLD. In addition, we notice that SLBZS still markedly reversed the negative effects of a high-fat diet on liver weight, body weight and some biochemical parameters even though SIRT1 expression was inhibited by EX 527. These results suggest that, in addition to SIRT1 activation, there may be other important mechanisms by which SLBZS regulates whole-body metabolism. Future studies are needed to investigate the mechanisms of SLBZS on other organs.

Interestingly, SIRT1 inhibition by EX 527 had no obvious negative effects on some body and biochemical parameters in HFD+EX group compared with those in HFD group. These findings may reflect the fact that the effect of SIRT1 inhibition is still controversial [28]. Although some studies have reported that SIRT1 inhibition in the liver can contribute to hepatic steatosis [29,30], there is some evidence that SIRT1 inhibition in the liver or adipocytes can enhance insulin sensitivity in animals exposed to a high-fat diet [31,32]. Moreover, it has been reported that SIRT1 inhibition could not significantly change the hepatic lipid content in rats fed a high-fat diet [28], which was partly consistent with our study. Taken together, our data indicated that the beneficial effects of SLBZS were partly diminished in the presence of the SIRT1 inhibitor EX 527. However, the exact mechanisms of SIRT1 inhibition in different pathological conditions remain controversial and warrant further clarification.

In recent years, the study of lipids has drawn much attention due to their essential roles in cellular function as well as to the great progress of analytical techniques and applications [13,33]. Lipidomics has emerged as an independent field of research that differs from metabolomics due to the diversity of lipid structures and functions [7]. Recently, accumulating studies applied lipidomics to investigate the mechanism of TCM on many diseases [34,35]. In the present study, we employed untargeted lipidomics to further investigate the mechanism of SLBZS on hepatic lipid metabolism in NAFLD rats. Our results from the multivariate analysis showed that high-fat feeding resulted in marked lipid alterations in the liver, which is in agreement with other studies in mice [36,37]. As expected, HFD+SL group was well separated from HFD group, indicating that SLBZS administration successfully induced lipid alterations in the liver. Moreover, we found that HFD+EX and HFD+EX+SL groups were separated from HFD group. This finding suggests that SIRT1 inhibition by EX 527 could induce marked lipid alterations in the livers of rats fed a high-fat diet.

It has been demonstrated that NAFLD is associated with numerous changes in the lipid composition of the human liver [38]. The lipidomic analysis of human liver revealed that TG levels were increased, whereas PC levels were decreased in NAFLD patients. Similarly, our results showed that TG species were significantly increased in HFD group, whereas most PC species were significantly decreased. The increased hepatic levels of TG species in NAFLD rats are in agreement with the fact that the excess TG accumulation in hepatocytes is one of the hallmarks of NAFLD [39]. However, SLBZS administration significantly reduced hepatic levels of 7 TG species in rats fed a high-fat diet. Moreover, pathway analysis showed that SLBZS administration had a great impact on glycerolipid metabolism. Glycerolipid is characterized by the presence of a glycerol molecule esterified with fatty acids, such as diacylglycerol (DG) and TG [40]. The glycerolipid/free fatty acid cycle in glycerolipid metabolism allows detoxification of lipids via lipolysis and fatty acid β-oxidation in the face of hyperlipidemia, thereby preventing hepatic steatosis [41]. Therefore, the glycerolipid/free fatty acid cycle may be implicated in the mechanism by which SLBZS regulates glycerolipid metabolism. In addition, it is widely believed that SIRT1 regulates hepatic de novo lipogenesis and fatty acid β-oxidation by regulating transcription factors such as sterol regulatory element binding protein-1c (SREBP-1c), carbohydrate response element binding protein (ChREBP), and peroxisome proliferator-activated receptor alpha (PPARα) [17,39]. Moreover, it has been proposed that SIRT1 activation could enhance the glycerolipid/free fatty acid cycle in glycerolipid metabolism [42]. This evidence indicates that SIRT1 may play important roles in glycerolipid metabolism. In our study, the effect of SLBZS on TG species was markedly diminished following SIRT1 inhibition by EX 527. Therefore, it seems likely that the beneficial effect of SLBZS on disturbed glycerolipid metabolism is related to SIRT1 activation in the liver.

PC is a hydrophilic lipid required for packaging and export of neutral lipids, such as TGs, in very-low-density lipoprotein (VLDL). Thus, impaired PC biosynthesis reduces VLDL synthesis and secretion, thereby contributing to the development of NAFLD [40]. In addition to PC species, we also found that many other glycerophospholipids, such as PE, PI, and CL species, were significantly decreased in HFD group. These findings are consistent with the concept that glycerophospholipids play several important roles in the progression of NAFLD [40,43]. However, although accumulating studies have demonstrated that some glycerophospholipid species are probable modulators of metabolic diseases, little is known about how different glycerophospholipids affect the organism’s susceptibility to metabolic comorbidities [43]. Interestingly, in our study, we observed that several glycerophospholipids, such as PC(16:0/18:1), PE(18:0/20:3), and PI(18:0/20:3), were significantly increased in HFD group, which seems contrary to most glycerophospholipids. These findings may indicate that the specific functions of different glycerophospholipids still require further clarification. Moreover, we observed that the decreases in many glycerophospholipids in the livers of rats fed a high-fat diet were effectively reversed by SLBZS administration. Consistently, pathway analysis results showed that glycerophospholipid metabolism was closely involved in the effect of SLBZS on hepatic lipid metabolism. However, the effect of SLBZS on glycerophospholipids was partly diminished by the SIRT1 inhibitor EX 527. It has been reported that hepatic deletion of SIRT1 could reduce mitochondrial glycerol-3-phosphate acyltransferase (GPAT) expression, which plays essential roles in glycerophospholipid synthesis [44,45]. Accordingly, it is possible that hepatic SIRT1 may be involved in the effect of SLBZS on glycerophospholipid metabolism. Intriguingly, we found that several glycerophospholipid species were still significantly changed following SLBZS administration despite SIRT1 inhibition by EX 527. This finding may imply the possibility that, in addition to SIRT1 activation, there are other pathways involved in the mechanism whereby SLBZS regulates glycerophospholipid metabolism, which warrants further investigation.

In addition to glycerophospholipids and glycerolipids, some sphingolipids have been reported to play potential roles in the development and progression of NAFLD [40,46]. Our results showed that several Cer and SM species were significantly changed by high-fat feeding, which was in agreement with other studies [36,47]. Moreover, we found that SLBZS administration reversed the abnormal hepatic levels of some sphingolipids, including Cer(d18:1/23:0) and SM(d16:1/18:0). This finding indicated the beneficial effect of SLBZS on sphingolipid metabolism. It has been proposed that many enzymes involved in sphingolipid synthesis may be potential therapeutic targets for the treatment of metabolic disorders [43]. Therefore, it is tempting to investigate the effects of SLBZS on the enzymes involved in sphingolipid synthesis in the future. Additionally, our results indicated that SIRT1 inhibition by EX 527 effectively diminished the effect of SLBZS on sphingolipids. It is possible that the role of SIRT1 in sphingolipid metabolism may be associated with the bidirectional crosstalk between sphingolipid metabolism and glycerophospholipid metabolism [48], but more studies are needed to clarify the interplay between SIRT1 and sphingolipid metabolism.

## 4. Materials and Methods

### 4.1. Animals and Drugs

A total of 40 specific-pathogen-free male Wistar rats (SCXK (Lu) 2014-0007), weighing 170 ± 10 g, were purchased from Pengyue Laboratory Animal Breeding Center (Jinan, China). All rats were maintained under controlled temperature (20–24 °C) and humidity (50–60%) and kept on a 12 h light/12-h dark cycle with normal food and water ad libitum. SLBZS consists of ten traditional Chinese medicines. The SLBZS used in the present study were in the form of formula granules and manufactured by Jiangyin Tian Jiang Pharmaceutical Co., Ltd. (Jiangyin, China) in line with Chinese Pharmacopeia (2015 version). The detailed information of SLBZS is listed in Supplementary Table S1. The quality evaluation of SLBZS was performed by liquid chromatography-mass spectrometry (LC-MS) as previously described [14,49]. The typical LC-MS chromatogram of SLBZS is shown in Supplementary Figure S1. The formula granules were dissolved in distilled water prior to the experiment.

### 4.2. Experimental Design

After 1 week of acclimatization, rats were randomly distributed into 5 groups (8 rats per group), namely, normal control (NC) group, high-fat diet (HFD) group, high-fat diet plus SLBZS (HFD + SL) group, high-fat diet plus EX 527 (HFD + EX) group, and high-fat diet plus EX 527 and SLBZS (HFD + EX + SL) group. Rats in NC group were fed a normal diet containing 13% of energy as fat, 22% of energy as protein and 65% of energy as carbohydrate (Trophic Animal Feed, Nantong, China), while rats in the other groups were fed a high-fat diet containing 37% of energy as fat, 22% of energy as protein and 41% of energy as carbohydrate (Trophic Animal Feed, Nantong, China) for 12 weeks. From the beginning of week 7, SLBZS in distilled water was given to rats in HFD + SL group and HFD + EX + SL group at a dose of 30 g/kg/day by oral gavage, while rats in the other groups received oral gavage of an equal volume of distilled water. This dosage of SLBZS used in the study was based on our previous studies [14,50]. EX 527 (a SIRT1-selective inhibitor, Selleck Chemicals, Houston, TX, USA) dissolved in vehicle (5% DMSO + 30% PEG 300 + dd H2O) was given to rats in HFD + EX group and HFD + EX + SL group by intraperitoneal injection at a dose of 5 mg/kg every other day 30 min before SLBZS administration [51,52,53], while rats in the other groups received intraperitoneal injection of an equal volume of vehicle. At the end of week 12, all rats were sacrificed after the liver blood flow test as described below. Then, blood samples were obtained from the abdominal aorta and centrifuged at 1500× *g* for 10 min at 4 °C, and serum samples were collected and stored at −80 °C for biochemical analyses. The livers were immediately harvested and weighed to calculate the liver index (liver weight/body weight × 100%), snap-frozen in liquid nitrogen and stored at −80 °C for subsequent analyses. The animal experiment was performed in accordance with the Guiding Principles for Animal Experiments of Jinan University, and the study protocol was approved by Institutional Animal Care and Use Committee of Jinan University (Ethic approval number: 20180412-04).

### 4.3. Liver Blood Flow Test

After the last administration and overnight fasting, rats were anesthetized by intraperitoneal injection of 3% pentobarbital (0.2 mL/100 g) [50] and underwent laparotomy. Then, the blood flow of the rat liver was assessed using the moorFLPI-2 Full-Field Laser Perfusion Imager (Moor Instruments, Axminster, UK). The recording duration was 30 s. The manufacturer’s software (moorFLPI-2 Review version 5.0, Moor Instruments, Axminster, UK) was used to analyze the images. Regions of Interest (ROIs) were placed on the right median, left median and left liver lobes. The flux of the ROIs was averaged to obtain the liver blood flow of each rat liver [54]. The blood flow intensity is expressed as the perfusion unit (PU), which is related to the product of average speed and concentration of moving red blood cells in the tissue volume.

### 4.4. Histological Assessment

Liver tissues were fixed in 4% paraformaldehyde solution for 12 h, dehydrated in ethyl alcohol, dealcoholized in xylene, embedded in paraffin, and sliced to a thickness of 5 μm. After deparaffinization and rehydration, liver sections were stained with hematoxylin and eosin (HE). To assess liver fat deposition, frozen liver samples were embedded in optimum cutting temperature compound (Sakura Finetek, Torrance, CA, USA), and sliced at a thickness of 8 μm at −18 °C. Frozen liver sections were stained with Oil Red O solution (Jiancheng Technology, Nanjing, China) and stained with hematoxylin. All liver sections were observed under a light microscope (Leica Microsystems, Wetzlar, Germany). The percentage of Oil Red O-stained area was measured using ImageJ.

### 4.5. Biochemical Analysis

Serum levels of total cholesterol (TC), triglyceride (TG), alanine aminotransferase (ALT) and aspartate aminotransferase (AST) were assessed using an automatic biochemical analyzer (Chemray 240; Rayto Life and Analytical Sciences Co., Ltd., Shenzhen, China). Liver levels of TC and TG were assessed using corresponding commercial kits (Jiancheng Technology, Nanjing, China) following the manufacturer’s protocols.

### 4.6. Western Blotting Analysis

Western blotting was used to determine the protein expression of SIRT1 in the liver. GAPDH was used as an internal control. Total protein was extracted from liver tissue with RIPA lysis buffer (Beyotime, Shanghai, China), and the protein concentration was determined using a BCA protein assay kit (Beyotime, Shanghai, China). Equal amounts of protein from each group were separated on 6% SDS-PAGE gels and transferred to PVDF membranes. Membranes were blocked with 5% skim milk in 0.1% Tween-20/TBS for 1 h at room temperature and then incubated with SIRT1 antibody (1:1000; Cell Signaling Technology, Danvers, MA, USA) or GAPDH antibody (1:1000; Cell Signaling Technology, Danvers, MA, USA) overnight at 4 °C. After washing, the membranes were incubated with HRP-linked anti-rabbit secondary antibody (1:2000, Cell Signaling Technology, Danvers, MA, USA) for 1 h at room temperature. The protein bands were visualized with the ChemiDoc Imaging system (Bio-Rad Laboratories, Hercules, CA, USA), and densitometric analysis of band intensities was performed with ImageJ (version 1.51k, National Institutes of Health, Bethesda, MD, USA).

### 4.7. Lipidomic Analysis

For lipidomic analysis, liver samples were thawed at 4 °C, and ~40 mg was homogenized in 200 μL of ice-cold water and combined with 240 μL of ice-cold methanol. The mixed samples were added to 800 μL of methyl tert-butyl ether and then sonicated in a cold water bath for 20 min. The mixture was incubated for 30 min at room temperature and then centrifuged at 14,000× *g* for 15 min at 10 °C. The upper phase was collected and dried with nitrogen. Each dried residue was reconstituted in 400 μL of isopropanol-acetonitrile solution (9:1, *v*/*v*) and centrifuged at 14,000× *g* for 15 min at 10 °C. The supernatants were collected for UHPLC-MS analysis. Quality control (QC) samples for method validation were prepared by mixing equivalent aliquots of all the samples.

The UPLC analysis was performed using a Nexera UHPLC LC-30A System (Shimadzu, Kyoto, Japan). A Waters ACQUITY UPLC CSH C18 column (1.7 μm, 2.1 mm × 100 mm, Waters, Milford, MA, USA) was applied for all analyses. The mobile phase, delivered at a flow rate of 300 μL/min, consisted of acetonitrile-water solution (6:4, *v*/*v*) containing 10 mM ammonium formate (A) and acetonitrile-isopropanol solution (1:9, *v*/*v*) containing 10 mM ammonium formate (B). The column temperature was 45 °C, and the injection volume was 2 μL. The gradient conditions were as follows: 0–2 min, 30% B; 2–25 min 30–100% B; 25–35 min 30% B. The autosampler was maintained at 10 °C throughout the analysis.

The mass spectrometry analysis was performed on Q Exactive Orbitrap mass spectrometer (Thermo Fisher Scientific, San Jose, CA, USA) using electrospray ionization (ESI) in both positive and negative ion modes. The conditions of positive ion mode: Heater temperature, 300 °C; sheath gas flow rate, 45 arb; aux gas flow rate, 15 arb; sweep gas flow rate, 1 arb; spray voltage, 3.0 kV; capillary temperature, 350 °C; S-Lens RF level, 50%; scan range, 200–1800 *m*/*z*. The conditions of negative ion mode: Heater temperature, 300 °C; sheath gas flow rate, 45 arb; aux gas flow rate, 15 arb; sweep gas flow rate, 1 arb; spray voltage, 2.5 kV; capillary temperature, 350 °C; S-Lens RF level, 60%; scan range, 250–1800 *m*/*z*.

### 4.8. Lipidomic Data Processing

LipidSearch software version 4.1 (Thermo Fisher Scientific, San Jose, CA, USA) was used to preprocess the raw data obtained from UHPLC-MS analysis. The procedures included peak identification, lipid identification, peak extraction, peak alignment and quantification. The main settings were as follows: Precursor tolerance, 5 ppm; product tolerance, 5 ppm; product ion threshold, 5%. The normalized dataset was imported into SIMCA 14.1 (MKS Umetrics, Malmö, Sweden) for multivariate analysis. Principal component analysis (PCA), partial least squares-discriminant analysis (PLS-DA) and orthogonal partial least squares-discriminant analysis (OPLS-DA) were performed. The quality of the models was evaluated with the goodness of fit parameter R2 and the predictive ability parameter Q2. R2 and Q2 values close to 1 indicate the high quality of the models. The significantly changed lipids between the two groups were identified according to the variable importance in the projection (VIP), S-plot and *p* value (unpaired Student’s *t*-test). The lipids with both multivariate and univariate statistical significance (OPLS-DA VIP > 1.5 and *p* < 0.05) were considered to be differential lipid species. Pathway analysis was performed with an online software MetaboAnalyst (http://www.metaboanalyst.ca, version 4.0, Xia Lab, McGill University, Montreal, Canada), and the pathway with an impact value > 0.1 was considered as a potential target pathway. Cytoscape software (version 3.7.1, Cytoscape Consortium, San Diego, CA, USA) was used to visualize the metabolic networks of differential lipid species.

### 4.9. Statistical Analysis

Data are expressed as the mean ± standard deviation and were analyzed with SPSS 22.0 for Windows (IBM, Armonk, NY, USA). Differences among groups were analyzed using one-way ANOVA followed by post hoc Tukey’s test or Games-Howell test, as appropriate. A *p*-value < 0.05 was considered to be statistically significant.

## 5. Conclusions

In our study, an untargeted lipidomics approach was successfully employed to investigate the protective effects of SLBZS against NAFLD in rats. Our results demonstrated that SLBZS administration effectively alleviated NAFLD and abnormal lipid metabolism in rats fed a high-fat diet. The effects of SLBZS on hepatic lipid species were mainly associated with the regulation of glycerophospholipid and glycerolipid metabolism. In addition, the mechanism underlying the beneficial effects of SLBZS on hepatic lipid metabolism may be at least partly attributed to SIRT1 activation in the liver. However, further investigations are needed to clarify the specific functions of individual lipid species and elucidate the exact mechanisms of SLBZS on relevant metabolic pathways. Furthermore, our study using untargeted lipidomics provided new insights into the underlying mechanisms of SLBZS against NAFLD.

## Figures and Tables

**Table 1:** List of the differential lipid species of the five groups.

No.	Identified Lipids	Category	HFD/NC		HFD+SL/HFD		HFD+EX+SL/HFD	
			Fold	Trend	Fold	Trend	Fold	Trend
1	TG(16:0/18:1/20:1)	GL	106.05	↑ **	0.64	↓ **	0.97	↓
2	TG(16:0/18:1/20:4)	GL	12.99	↑ **	0.63	↓ **	0.9	↓
3	TG(16:0/18:2/22:4)	GL	22.08	↑ **	0.6	↓ **	0.9	↓
4	TG(18:0/16:0/18:1)	GL	76.77	↑ **	0.63	↓ **	0.99	↓
5	TG(18:1/18:1/18:1)	GL	52.39	↑ **	0.78	↓ *	0.98	↓
6	TG(18:1/18:1/20:3)	GL	22.58	↑ **	0.6	↓ *	0.89	↓
7	TG(20:1/18:1/18:1)	GL	89.85	↑ **	0.67	↓ **	1.03	↑
8	CL(18:2/18:2/18:2/18:2)	GP	0.38	↓ **	1.71	↑ **	1.26	↑ **
9	PC(16:0/18:1)	GP	2.12	↑ **	1.18	↑ *	1.1	↑
10	PC(16:0/18:2)	GP	0.63	↓ **	1.35	↑ **	1.14	↑
11	PC(18:0/20:3)	GP	1.54	↑ *	1.73	↑ **	1.4	↑
12	PC(18:1/18:1)	GP	0.79	↓ *	1.4	↑ **	1.18	↑
13	PC(34:2)	GP	0.66	↓ **	1.35	↑ **	1.12	↑
14	PC(36:2)	GP	0.8	↓ *	1.36	↑ **	1.16	↑
15	PC(36:3)	GP	0.29	↓ **	1.27	↑ *	1.11	↑
16	PC(36:4)	GP	0.29	↓ **	1.69	↑ **	1.16	↑
17	PC(38:6)	GP	0.32	↓ **	1.42	↑ *	1.08	↑
18	PC(40:6)	GP	0.35	↓ **	1.29	↑ *	1.09	↑
19	PE(16:0/18:1)	GP	0.63	↓ **	1.3	↑ **	1.09	↑
20	PE(16:1/18:1)	GP	0.46	↓ **	1.54	↑ **	1.17	↑ *
21	PE(18:0/20:3)	GP	1.68	↑ *	1.65	↑ **	1.26	↑
22	PE(18:0/20:4)	GP	0.78	↓ *	1.13	↑ *	1.03	↑
23	PE(18:1/22:5)	GP	0.49	↓ **	1.21	↑ *	1.07	↑
24	PE(34:2)	GP	0.44	↓ **	1.58	↑ **	1.15	↑
25	PE(36:2)	GP	0.68	↓ *	1.48	↑ **	1.08	↑ *
26	PI(16:0/22:4)	GP	0.82	↓ *	1.12	↑ *	1.06	↑
27	PI(18:0/20:3)	GP	2.03	↑ *	1.72	↑ **	1.28	↑
28	PS(39:3)	GP	0.37	↓ **	1.64	↑ **	1.21	↑
29	Cer(d18:1/23:0)	SP	0.31	↓ **	1.71	↑ **	0.96	↓
30	SM(d16:1/18:0)	SP	1.84	↑ *	0.64	↓ *	1.01	↑

GL: Glycerolipid. GP: Glycerophospholipid. SP: Sphingolipid. The up arrow (↑) denotes a relative increase, and the down arrow (↓) denotes a relative decrease. * *p* < 0.05, ** *p* < 0.01. The *p* value was calculated by the unpaired Student’s *t*-test.

**Table 2:** Metabolic pathway analysis with MetaboAnalyst from differential lipid species.

Pathway Name	Total	Hits	Impact	Raw *p*	FDR	−log(*p*)
Glycerophospholipid metabolism	30	3	0.254	0.000	0.024	8.137
Glycerolipid metabolism	18	1	0.107	0.087	1	2.446
Glycosylphosphatidylinositol(GPI)-anchor biosynthesis	14	1	0.044	0.068	1	2.689
Linoleic acid metabolism	5	1	0.000	0.025	1	3.699
alpha-Linolenic acid metabolism	9	1	0.000	0.044	1	3.120
Sphingolipid metabolism	21	1	0.000	0.100	1	2.298
Arachidonic acid metabolism	36	1	0.000	0.167	1	1.791

Total: The total number of metabolites in the pathway. Hits: The actually matched number from the differential lipid species. Impact: The pathway impact value calculated from pathway topology analysis. Raw *p*: Original *p* value calculated from the enrichment analysis. FDR: The *p* value adjusted using the false discovery rate.

## Supplementary Materials

The following are available online at https://www.mdpi.com/1420-3049/24/21/3943/s1, Figure S1: Typical LC-MS chromatogram of SLBZS. Peak 1: Ginsenoside Rb1, Peak 2: Ginsenoside Rh1, Peak 3: Atractylenolide III, Peak 4: Pachymic acid; Figure S2: Base peak intensity chromatograms of quality control samples in positive and negative ion modes; Figure S3: PCA score plot of all samples; Figure S4: Lipid numbers of different lipid classes identified in this study; Figure S5: Venn diagram and upset plot of the five groups; Figure S6: Metabolic networks of differential lipid species visualized in Cytoscape; Table S1: Detailed information of Shenling Baizhu San; Table S2: Differential lipid species in HFD group relative to NC group; Table S3: Differential lipid species in HFD + SL group relative to HFD group; Table S4: Differential lipid species in HFD + EX + SL group relative to HFD group.
