# Supplementary material for: Lipidomic Analysis of the Protective Effects of Shenling Baizhu San on Non-Alcoholic Fatty Liver Disease in Rats
Source: Molecules. 2019 Oct 31;24(21):3943. doi: 10.3390/molecules24213943 (PMC6864612; doi:10.3390/molecules24213943)
Supplement: Supplementary file 1 [file molecules-24-03943-s001.zip › Supplementary Tables.docx]

Table S1. Detailed information of Shenling Baizhu San.

| **Chinese name** | **Latin name** | **Family** | **Part used** | **Proportion** |
| --- | --- | --- | --- | --- |
| Renshen | *Panax ginseng* C. A. Mey. | Araliaceae | Root | 5 |
| Fuling | *Poria cocos* (Schw.) Wolf. | Polyporaceae | Sclerotium | 5 |
| Baizhu | *Atractylodes macrocephala* Koidz. | Asteraceae | Rhizome | 5 |
| Shanyao | *Dioscorea opposita* Thunb. | Dioscoreaceae | Rhizome | 5 |
| Baibiandou | *Dolichos lablab* L. | Fabaceae | Seed | 4 |
| Lianzi | *Nelumbo nucifera* Gaertn. | Nymphaeaceae | Seed | 3 |
| Yiyiren | *Coix lacryma-jobi* L. | Poaceae | Kernel | 3 |
| Zhigancao | *Glycyrrhiza uralensis* Fisch. ex DC. | Fabaceae | Root and rhizome | 3 |
| Jiegeng | *Platycodon grandiflorus* (Jacq.) A. DC. | Campanulaceae | Root | 2 |
| Sharen | *Amomum villosum* Lour. | Zingiberaceae | Fruit | 2 |

Table S2. Differential lipid species in HFD group relative to NC group.

| **No.** | **Lipid ion** | **Lipid group** | **Category** | **Ion formula** | **m/z** | **RT (min)** | **Fold** | **VIP** | **P value** |
| --- | --- | --- | --- | --- | --- | --- | --- | --- | --- |
| 1 | TG(16:0/16:0/18:1)+NH4 | TG(50:1)+NH4 | GL | C53 H104 O6 N1 | 850.79 | 23.17 | 31.15 | 3.32 | <0.01 |
| 2 | TG(16:0/16:0/18:3)+NH4 | TG(50:3)+NH4 | GL | C53 H100 O6 N1 | 846.75 | 21.45 | 23.67 | 2.51 | <0.01 |
| 3 | TG(16:0/16:1/18:1)+NH4 | TG(50:2)+NH4 | GL | C53 H102 O6 N1 | 848.77 | 22.35 | 11.74 | 3.35 | <0.01 |
| 4 | TG(16:0/17:1/18:1)+NH4 | TG(51:2)+NH4 | GL | C54 H104 O6 N1 | 862.79 | 22.77 | 44.97 | 1.62 | <0.01 |
| 5 | TG(16:0/18:1/18:1)+NH4 | TG(52:2)+NH4 | GL | C55 H106 O6 N1 | 876.80 | 23.16 | 41.59 | 8.95 | <0.01 |
| 6 | TG(16:0/18:1/18:2)+NH4 | TG(52:3)+NH4 | GL | C55 H104 O6 N1 | 874.79 | 22.46 | 8.26 | 8.10 | <0.01 |
| 7 | TG(16:0/18:1/18:3)+NH4 | TG(52:4)+NH4 | GL | C55 H102 O6 N1 | 872.77 | 21.59 | 2.63 | 3.69 | <0.01 |
| 8 | TG(16:0/18:1/20:1)+NH4 | TG(54:2)+NH4 | GL | C57 H110 O6 N1 | 904.83 | 23.83 | 106.05 | 4.34 | <0.01 |
| 9 | TG(16:0/18:1/20:4)+NH4 | TG(54:5)+NH4 | GL | C57 H104 O6 N1 | 898.79 | 22.18 | 12.99 | 2.88 | <0.01 |
| 10 | TG(16:0/18:2/22:4)+NH4 | TG(56:6)+NH4 | GL | C59 H106 O6 N1 | 924.80 | 22.17 | 22.08 | 3.05 | <0.01 |
| 11 | TG(17:0/18:1/18:1)+NH4 | TG(53:2)+NH4 | GL | C56 H108 O6 N1 | 890.82 | 23.51 | 71.23 | 1.53 | <0.01 |
| 12 | TG(18:0/16:0/18:1)+NH4 | TG(52:1)+NH4 | GL | C55 H108 O6 N1 | 878.82 | 23.84 | 76.77 | 3.11 | <0.01 |
| 13 | TG(18:1/17:1/18:1)+NH4 | TG(53:3)+NH4 | GL | C56 H106 O6 N1 | 888.80 | 22.87 | 26.62 | 1.56 | <0.01 |
| 14 | TG(18:1/18:1/18:1)+NH4 | TG(54:3)+NH4 | GL | C57 H108 O6 N1 | 902.82 | 23.20 | 52.39 | 6.95 | <0.01 |
| 15 | TG(18:1/18:1/18:2)+NH4 | TG(54:4)+NH4 | GL | C57 H106 O6 N1 | 900.80 | 22.47 | 14.10 | 5.66 | <0.01 |
| 16 | TG(18:1/18:1/20:3)+NH4 | TG(56:5)+NH4 | GL | C59 H108 O6 N1 | 926.82 | 23.01 | 22.58 | 1.81 | <0.01 |
| 17 | TG(18:1/18:2/18:2)+NH4 | TG(54:5)+NH4 | GL | C57 H104 O6 N1 | 898.79 | 21.61 | 7.27 | 4.42 | <0.01 |
| 18 | TG(18:1/18:2/20:3)+NH4 | TG(56:6)+NH4 | GL | C59 H106 O6 N1 | 924.80 | 21.85 | 11.53 | 1.89 | <0.01 |
| 19 | TG(20:1/18:1/18:1)+NH4 | TG(56:3)+NH4 | GL | C59 H112 O6 N1 | 930.85 | 23.79 | 89.85 | 2.50 | <0.01 |
| 20 | TG(54:5)+H | TG(54:5)+H | GL | C57 H101 O6 | 881.76 | 23.10 | 6.80 | 1.92 | <0.01 |
| 21 | CL(18:2/18:2/18:2/18:2)-H | CL(72:8)-H | GP | C81 H141 O17 P2 | 1447.96 | 18.52 | 0.38 | 1.59 | <0.01 |
| 22 | LPC(20:4)+H | LPC(20:4)+H | GP | C28 H51 O7 N1 P1 | 544.34 | 2.44 | 0.32 | 1.59 | <0.01 |
| 23 | LPE(20:4)-H | LPE(20:4)-H | GP | C25 H43 O7 N1 P1 | 500.28 | 2.61 | 0.41 | 1.73 | <0.01 |
| 24 | LPE(22:6)-H | LPE(22:6)-H | GP | C27 H43 O7 N1 P1 | 524.28 | 2.49 | 0.22 | 1.75 | <0.01 |
| 25 | PC(16:0/18:1)+HCOO | PC(34:1)+HCOO | GP | C43 H83 O10 N1 P1 | 804.58 | 12.31 | 2.12 | 4.26 | <0.01 |
| 26 | PC(16:0/18:2)+HCOO | PC(34:2)+HCOO | GP | C43 H81 O10 N1 P1 | 802.56 | 11.51 | 0.63 | 2.03 | <0.01 |
| 27 | PC(16:0/22:6)+HCOO | PC(38:6)+HCOO | GP | C47 H81 O10 N1 P1 | 850.56 | 11.00 | 0.35 | 3.12 | <0.01 |
| 28 | PC(18:0/18:1)+HCOO | PC(36:1)+HCOO | GP | C45 H87 O10 N1 P1 | 832.61 | 13.40 | 2.00 | 2.54 | <0.01 |
| 29 | PC(18:0/20:3)+HCOO | PC(38:3)+HCOO | GP | C47 H87 O10 N1 P1 | 856.61 | 12.78 | 1.54 | 1.58 | 0.030 |
| 30 | PC(18:0/20:4)+HCOO | PC(38:4)+HCOO | GP | C47 H85 O10 N1 P1 | 854.59 | 12.36 | 0.65 | 2.26 | <0.01 |
| 31 | PC(18:1/18:1)+HCOO | PC(36:2)+HCOO | GP | C45 H85 O10 N1 P1 | 830.59 | 12.54 | 0.79 | 1.67 | 0.010 |
| 32 | PC(18:2/20:4)+HCOO | PC(38:6)+HCOO | GP | C47 H81 O10 N1 P1 | 850.56 | 10.54 | 0.38 | 1.98 | <0.01 |
| 33 | PC(32:0)+H | PC(32:0)+H | GP | C40 H81 O8 N1 P1 | 734.57 | 12.16 | 0.77 | 1.57 | 0.029 |
| 34 | PC(34:1)+H | PC(34:1)+H | GP | C42 H83 O8 N1 P1 | 760.59 | 12.23 | 2.12 | 1.89 | <0.01 |
| 35 | PC(34:2)+H | PC(34:2)+H | GP | C42 H81 O8 N1 P1 | 758.57 | 11.43 | 0.66 | 7.00 | <0.01 |
| 36 | PC(36:1)+Na | PC(36:1)+Na | GP | C44 H86 O8 N1 P1 Na1 | 810.60 | 12.26 | 0.64 | 8.39 | <0.01 |
| 37 | PC(36:2)+H | PC(36:2)+H | GP | C44 H85 O8 N1 P1 | 786.60 | 12.46 | 0.80 | 5.57 | 0.020 |
| 38 | PC(36:3)+Na | PC(36:3)+Na | GP | C44 H82 O8 N1 P1 Na1 | 806.57 | 10.91 | 0.29 | 5.14 | <0.01 |
| 39 | PC(36:4)+H | PC(36:4)+H | GP | C44 H81 O8 N1 P1 | 782.57 | 10.66 | 0.29 | 2.38 | <0.01 |
| 40 | PC(37:4)+H | PC(37:4)+H | GP | C45 H83 O8 N1 P1 | 796.59 | 11.74 | 0.20 | 2.38 | <0.01 |
| 41 | PC(37:6)+H | PC(37:6)+H | GP | C45 H79 O8 N1 P1 | 792.55 | 12.24 | 0.50 | 1.99 | <0.01 |
| 42 | PC(38:6)+H | PC(38:6)+H | GP | C46 H81 O8 N1 P1 | 806.57 | 10.46 | 0.32 | 3.18 | <0.01 |
| 43 | PC(39:4)+H | PC(39:4)+H | GP | C47 H87 O8 N1 P1 | 824.62 | 12.62 | 0.15 | 1.54 | <0.01 |
| 44 | PC(40:6)+H | PC(40:6)+H | GP | C48 H85 O8 N1 P1 | 834.60 | 11.97 | 0.35 | 4.45 | <0.01 |
| 45 | PC(40:6)+Na | PC(40:6)+Na | GP | C48 H84 O8 N1 P1 Na1 | 856.58 | 11.97 | 0.44 | 1.58 | <0.01 |
| 46 | PC(40:8)+H | PC(40:8)+H | GP | C48 H81 O8 N1 P1 | 830.57 | 10.20 | 0.30 | 1.84 | <0.01 |
| 47 | PE(16:0/18:1)+Na | PE(34:1)+Na | GP | C39 H76 O8 N1 P1 Na1 | 740.52 | 11.53 | 0.63 | 2.55 | <0.01 |
| 48 | PE(16:0/22:6)-H | PE(38:6)-H | GP | C43 H73 O8 N1 P1 | 762.51 | 11.34 | 0.46 | 2.83 | <0.01 |
| 49 | PE(16:1/18:1)-H | PE(34:2)-H | GP | C39 H73 O8 N1 P1 | 714.51 | 11.80 | 0.46 | 2.96 | <0.01 |
| 50 | PE(18:0/18:1)+Na | PE(36:1)+Na | GP | C41 H80 O8 N1 P1 Na1 | 768.55 | 12.57 | 0.76 | 4.09 | <0.01 |
| 51 | PE(18:0/18:1)-H | PE(36:1)-H | GP | C41 H79 O8 N1 P1 | 744.55 | 13.72 | 2.29 | 1.78 | <0.01 |
| 52 | PE(18:0/20:3)-H | PE(38:3)-H | GP | C43 H79 O8 N1 P1 | 768.55 | 13.09 | 1.68 | 1.52 | <0.01 |
| 53 | PE(18:0/20:4)-H | PE(38:4)-H | GP | C43 H77 O8 N1 P1 | 766.54 | 12.68 | 0.78 | 2.15 | <0.01 |
| 54 | PE(18:1/18:2)+Na | PE(36:3)+Na | GP | C41 H76 O8 N1 P1 Na1 | 764.52 | 11.21 | 0.52 | 2.49 | <0.01 |
| 55 | PE(18:1/22:5)-H | PE(40:6)-H | GP | C45 H77 O8 N1 P1 | 790.54 | 12.34 | 0.49 | 2.33 | <0.01 |
| 56 | PE(19:0/20:4)+H | PE(39:4)+H | GP | C44 H81 O8 N1 P1 | 782.57 | 11.23 | 0.66 | 7.43 | <0.01 |
| 57 | PE(34:2)+H | PE(34:2)+H | GP | C39 H75 O8 N1 P1 | 716.52 | 11.72 | 0.44 | 2.47 | <0.01 |
| 58 | PE(36:2)+H | PE(36:2)+H | GP | C41 H79 O8 N1 P1 | 744.55 | 12.76 | 0.68 | 2.14 | <0.01 |
| 59 | PI(16:0/22:4)-H | PI(38:4)-H | GP | C47 H82 O13 N0 P1 | 885.55 | 11.18 | 0.82 | 2.82 | <0.01 |
| 60 | PI(18:0/20:3)-H | PI(38:3)-H | GP | C47 H84 O13 N0 P1 | 887.57 | 11.58 | 2.03 | 2.55 | <0.01 |
| 61 | PI(18:0/20:4)+NH4 | PI(38:4)+NH4 | GP | C47 H87 O13 N1 P1 | 904.59 | 11.10 | 0.64 | 1.60 | <0.01 |
| 62 | PS(39:3)-H | PS(39:3)-H | GP | C45 H81 O10 N1 P1 | 826.56 | 10.73 | 0.37 | 1.53 | <0.01 |
| 63 | PS(43:5)-H | PS(43:5)-H | GP | C49 H85 O10 N1 P1 | 878.59 | 12.04 | 0.36 | 2.68 | <0.01 |
| 64 | Cer(d18:1/23:0)+HCOO | Cer(d41:1)+HCOO | SP | C42 H82 O5 N1 | 680.62 | 17.00 | 0.31 | 1.83 | <0.01 |
| 65 | SM(d16:1/18:0)+HCOO | SM(d34:1)+HCOO | SP | C40 H80 O8 N2 P1 | 747.57 | 11.21 | 1.84 | 2.26 | 0.010 |
| 66 | SM(d22:1/20:1)+HCOO | SM(d42:2)+HCOO | SP | C48 H94 O8 N2 P1 | 857.68 | 14.54 | 1.34 | 1.75 | 0.015 |
| 67 | SM(d42:1)+H | SM(d42:1)+H | SP | C47 H96 O6 N2 P1 | 815.70 | 15.79 | 0.67 | 2.04 | <0.01 |

GL: glycerolipid. GP: glycerophospholipid. SP: sphingolipid.

Table S3. Differential lipid species in HFD+SL group relative to HFD group.

| **No.** | **Lipid ion** | **Lipid group** | **Category** | **Ion formula** | **m/z** | **RT (min)** | **Fold** | **VIP** | **P value** |
| --- | --- | --- | --- | --- | --- | --- | --- | --- | --- |
| 1 | TG(16:0/18:1/20:1)+NH4 | TG(54:2)+NH4 | GL | C57 H110 O6 N1 | 904.83 | 23.83 | 0.64 | 5.11 | <0.01 |
| 2 | TG(16:0/18:1/20:4)+NH4 | TG(54:5)+NH4 | GL | C57 H104 O6 N1 | 898.79 | 22.18 | 0.63 | 3.65 | <0.01 |
| 3 | TG(16:0/18:2/22:4)+NH4 | TG(56:6)+NH4 | GL | C59 H106 O6 N1 | 924.80 | 22.17 | 0.60 | 3.89 | <0.01 |
| 4 | TG(18:0/16:0/18:1)+NH4 | TG(52:1)+NH4 | GL | C55 H108 O6 N1 | 878.82 | 23.84 | 0.63 | 3.72 | <0.01 |
| 5 | TG(18:0/18:0/18:1)+NH4 | TG(54:1)+NH4 | GL | C57 H112 O6 N1 | 906.85 | 24.40 | 0.54 | 1.76 | <0.01 |
| 6 | TG(18:0/18:1/20:1)+NH4 | TG(56:2)+NH4 | GL | C59 H114 O6 N1 | 932.86 | 24.37 | 0.59 | 1.74 | <0.01 |
| 7 | TG(18:1/18:1/18:1)+NH4 | TG(54:3)+NH4 | GL | C57 H108 O6 N1 | 902.82 | 23.20 | 0.78 | 5.75 | 0.047 |
| 8 | TG(18:1/18:1/20:3)+NH4 | TG(56:5)+NH4 | GL | C59 H108 O6 N1 | 926.82 | 23.01 | 0.60 | 2.28 | 0.010 |
| 9 | TG(20:1/18:1/18:1)+NH4 | TG(56:3)+NH4 | GL | C59 H112 O6 N1 | 930.85 | 23.79 | 0.67 | 2.77 | <0.01 |
| 10 | CL(18:2/18:2/18:2/18:2)-H | CL(72:8)-H | GP | C81 H141 O17 P2 | 1447.96 | 18.52 | 1.71 | 2.10 | <0.01 |
| 11 | PC(16:0/18:1)+HCOO | PC(34:1)+HCOO | GP | C43 H83 O10 N1 P1 | 804.58 | 12.31 | 1.18 | 2.00 | 0.010 |
| 12 | PC(16:0/18:2)+HCOO | PC(34:2)+HCOO | GP | C43 H81 O10 N1 P1 | 802.56 | 11.51 | 1.35 | 3.85 | <0.01 |
| 13 | PC(18:0/20:3)+HCOO | PC(38:3)+HCOO | GP | C47 H87 O10 N1 P1 | 856.61 | 12.78 | 1.73 | 2.92 | <0.01 |
| 14 | PC(18:0p/16:0)+HCOO | PC(34:0p)+HCOO | GP | C43 H85 O9 N1 P1 | 790.60 | 12.98 | 0.54 | 1.82 | 0.048 |
| 15 | PC(18:1/18:1)+HCOO | PC(36:2)+HCOO | GP | C45 H85 O10 N1 P1 | 830.59 | 12.54 | 1.40 | 4.33 | <0.01 |
| 16 | PC(18:1/18:2)+HCOO | PC(36:3)+HCOO | GP | C45 H83 O10 N1 P1 | 828.58 | 11.59 | 1.56 | 2.39 | <0.01 |
| 17 | PC(34:2)+H | PC(34:2)+H | GP | C42 H81 O8 N1 P1 | 758.57 | 11.43 | 1.35 | 7.03 | <0.01 |
| 18 | PC(35:5)+H | PC(35:5)+H | GP | C43 H77 O8 N1 P1 | 766.54 | 11.60 | 1.33 | 1.77 | <0.01 |
| 19 | PC(36:2)+H | PC(36:2)+H | GP | C44 H85 O8 N1 P1 | 786.60 | 12.46 | 1.36 | 6.72 | <0.01 |
| 20 | PC(36:3)+Na | PC(36:3)+Na | GP | C44 H82 O8 N1 P1 Na1 | 806.57 | 10.91 | 1.27 | 2.48 | 0.011 |
| 21 | PC(36:4)+H | PC(36:4)+H | GP | C44 H81 O8 N1 P1 | 782.57 | 10.66 | 1.69 | 1.86 | <0.01 |
| 22 | PC(38:3)+H | PC(38:3)+H | GP | C46 H87 O8 N1 P1 | 812.62 | 12.71 | 1.72 | 3.97 | <0.01 |
| 23 | PC(38:5)+H | PC(38:5)+H | GP | C46 H83 O8 N1 P1 | 808.59 | 11.62 | 1.22 | 1.66 | 0.033 |
| 24 | PC(38:6)+H | PC(38:6)+H | GP | C46 H81 O8 N1 P1 | 806.57 | 10.46 | 1.42 | 2.07 | 0.029 |
| 25 | PC(40:6)+H | PC(40:6)+H | GP | C48 H85 O8 N1 P1 | 834.60 | 11.97 | 1.29 | 2.56 | 0.011 |
| 26 | PE(16:0/18:1)+Na | PE(34:1)+Na | GP | C39 H76 O8 N1 P1 Na1 | 740.52 | 11.53 | 1.30 | 2.32 | <0.01 |
| 27 | PE(16:0/20:3)-H | PE(36:3)-H | GP | C41 H75 O8 N1 P1 | 740.52 | 11.88 | 1.55 | 2.69 | <0.01 |
| 28 | PE(16:0/22:5)-H | PE(38:5)-H | GP | C43 H75 O8 N1 P1 | 764.52 | 12.00 | 1.58 | 1.90 | <0.01 |
| 29 | PE(16:1/18:1)-H | PE(34:2)-H | GP | C39 H73 O8 N1 P1 | 714.51 | 11.80 | 1.54 | 4.18 | <0.01 |
| 30 | PE(18:0/18:2)-H | PE(36:2)-H | GP | C41 H77 O8 N1 P1 | 742.54 | 12.85 | 1.43 | 3.91 | <0.01 |
| 31 | PE(18:0/20:3)-H | PE(38:3)-H | GP | C43 H79 O8 N1 P1 | 768.55 | 13.09 | 1.65 | 2.47 | <0.01 |
| 32 | PE(18:0/20:4)-H | PE(38:4)-H | GP | C43 H77 O8 N1 P1 | 766.54 | 12.68 | 1.13 | 4.62 | 0.045 |
| 33 | PE(18:0/20:5)-H | PE(38:5)-H | GP | C43 H75 O8 N1 P1 | 764.52 | 11.69 | 1.31 | 2.14 | <0.01 |
| 34 | PE(18:1/18:3)-H | PE(36:4)-H | GP | C41 H73 O8 N1 P1 | 738.51 | 11.62 | 1.27 | 2.14 | <0.01 |
| 35 | PE(18:1/22:5)-H | PE(40:6)-H | GP | C45 H77 O8 N1 P1 | 790.54 | 12.34 | 1.21 | 1.61 | 0.019 |
| 36 | PE(34:2)+H | PE(34:2)+H | GP | C39 H75 O8 N1 P1 | 716.52 | 11.72 | 1.58 | 2.38 | <0.01 |
| 37 | PE(36:2)+H | PE(36:2)+H | GP | C41 H79 O8 N1 P1 | 744.55 | 12.76 | 1.48 | 2.60 | <0.01 |
| 38 | PI(16:0/22:4)-H | PI(38:4)-H | GP | C47 H82 O13 N0 P1 | 885.55 | 11.18 | 1.12 | 4.30 | 0.033 |
| 39 | PI(18:0/20:3)-H | PI(38:3)-H | GP | C47 H84 O13 N0 P1 | 887.57 | 11.58 | 1.72 | 4.03 | <0.01 |
| 40 | PI(18:1/18:1)-H | PI(36:2)-H | GP | C45 H82 O13 N0 P1 | 861.55 | 11.39 | 1.52 | 1.73 | 0.032 |
| 41 | PI(18:2/18:2)-H | PI(36:4)-H | GP | C45 H78 O13 N0 P1 | 857.52 | 10.12 | 1.67 | 2.23 | <0.01 |
| 42 | PS(39:3)-H | PS(39:3)-H | GP | C45 H81 O10 N1 P1 | 826.56 | 10.73 | 1.64 | 1.70 | <0.01 |
| 43 | Cer(d18:1/22:0)+HCOO | Cer(d40:1)+HCOO | SP | C41 H80 O5 N1 | 666.60 | 16.31 | 1.49 | 1.65 | <0.01 |
| 44 | Cer(d18:1/23:0)+HCOO | Cer(d41:1)+HCOO | SP | C42 H82 O5 N1 | 680.62 | 17.00 | 1.71 | 1.91 | <0.01 |
| 45 | Cer(d18:1/24:0)+HCOO | Cer(d42:1)+HCOO | SP | C43 H84 O5 N1 | 694.64 | 17.49 | 1.54 | 1.91 | <0.01 |
| 46 | Cer(d18:1/25:0)+HCOO | Cer(d43:1)+HCOO | SP | C44 H86 O5 N1 | 708.65 | 18.15 | 1.50 | 1.55 | <0.01 |
| 47 | SM(d16:1/18:0)+HCOO | SM(d34:1)+HCOO | SP | C40 H80 O8 N2 P1 | 747.57 | 11.21 | 0.64 | 4.14 | 0.036 |

GL: glycerolipid. GP: glycerophospholipid. SP: sphingolipid.

Table S4. Differential lipid species in HFD+EX+SL group relative to HFD group.

| **No.** | **Lipid ion** | **Lipid group** | **Category** | **Ion formula** | **m/z** | **RT (min)** | **Fold** | **VIP** | **P value** |
| --- | --- | --- | --- | --- | --- | --- | --- | --- | --- |
| 1 | CL(18:2/18:2/18:2/18:2)-H | CL(72:8)-H | GP | C81 H141 O17 P2 | 1447.96 | 18.52 | 1.26 | 1.56 | <0.01 |
| 2 | PC(18:0/20:3)+HCOO | PC(38:3)+HCOO | GP | C47 H87 O10 N1 P1 | 856.61 | 12.78 | 1.40 | 4.57 | 0.049 |
| 3 | PC(18:1/18:2)+HCOO | PC(36:3)+HCOO | GP | C45 H83 O10 N1 P1 | 828.58 | 11.59 | 1.31 | 3.77 | 0.019 |
| 4 | PC(20:1/18:1)+HCOO | PC(38:2)+HCOO | GP | C47 H89 O10 N1 P1 | 858.62 | 13.50 | 1.59 | 1.62 | 0.034 |
| 5 | PC(34:2)+H | PC(34:2)+H | GP | C42 H81 O8 N1 P1 | 758.57 | 11.43 | 1.12 | 5.53 | 0.022 |
| 6 | PC(38:3)+H | PC(38:3)+H | GP | C46 H87 O8 N1 P1 | 812.62 | 12.71 | 1.39 | 4.75 | <0.01 |
| 7 | PI(18:1/18:2)-H | PI(36:3)-H | GP | C45 H80 O13 N0 P1 | 859.53 | 10.38 | 1.70 | 2.03 | <0.01 |
| 8 | PI(18:2/18:2)-H | PI(36:4)-H | GP | C45 H78 O13 N0 P1 | 857.52 | 10.12 | 1.33 | 2.99 | <0.01 |

GP: glycerophospholipid.
