# Supplementary figures and images for: Lipidomic Analysis of the Protective Effects of Shenling Baizhu San on Non-Alcoholic Fatty Liver Disease in Rats
Source: Molecules. 2019 Oct 31;24(21):3943. doi: 10.3390/molecules24213943 (PMC6864612; doi:10.3390/molecules24213943)

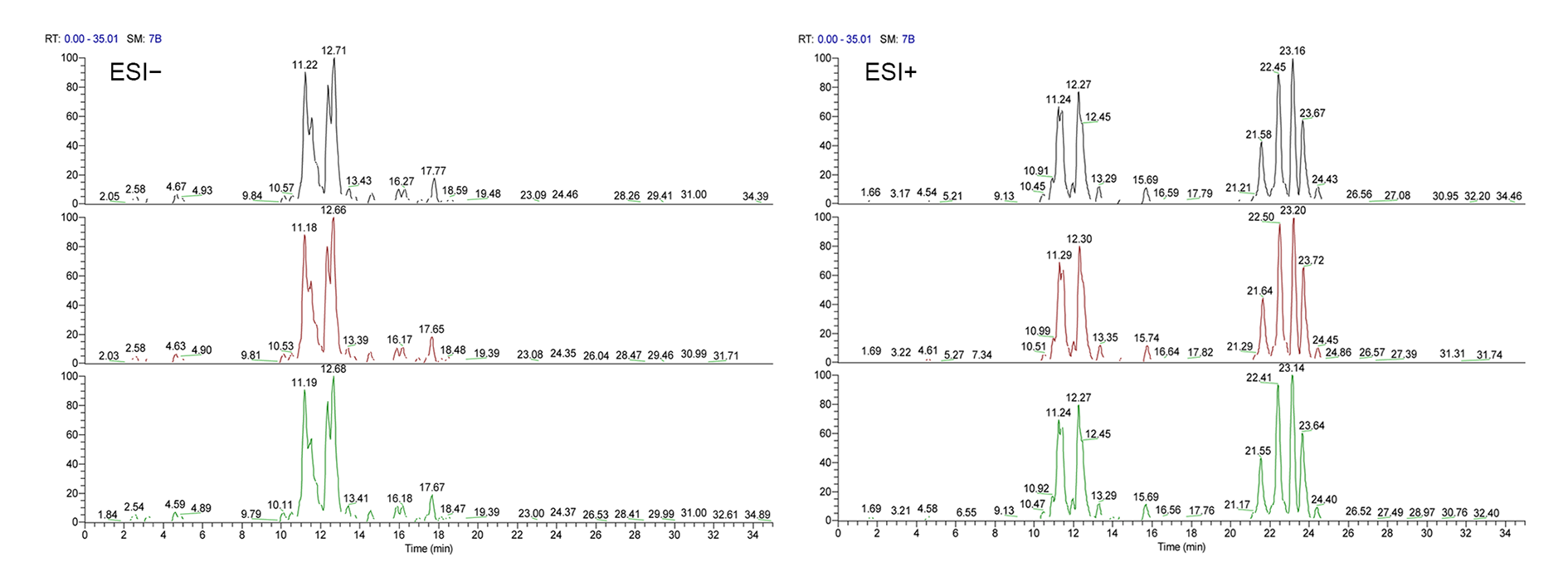

Supplement: Supplementary file 1 [file molecules-24-03943-s001.zip › Figure S2.tif]

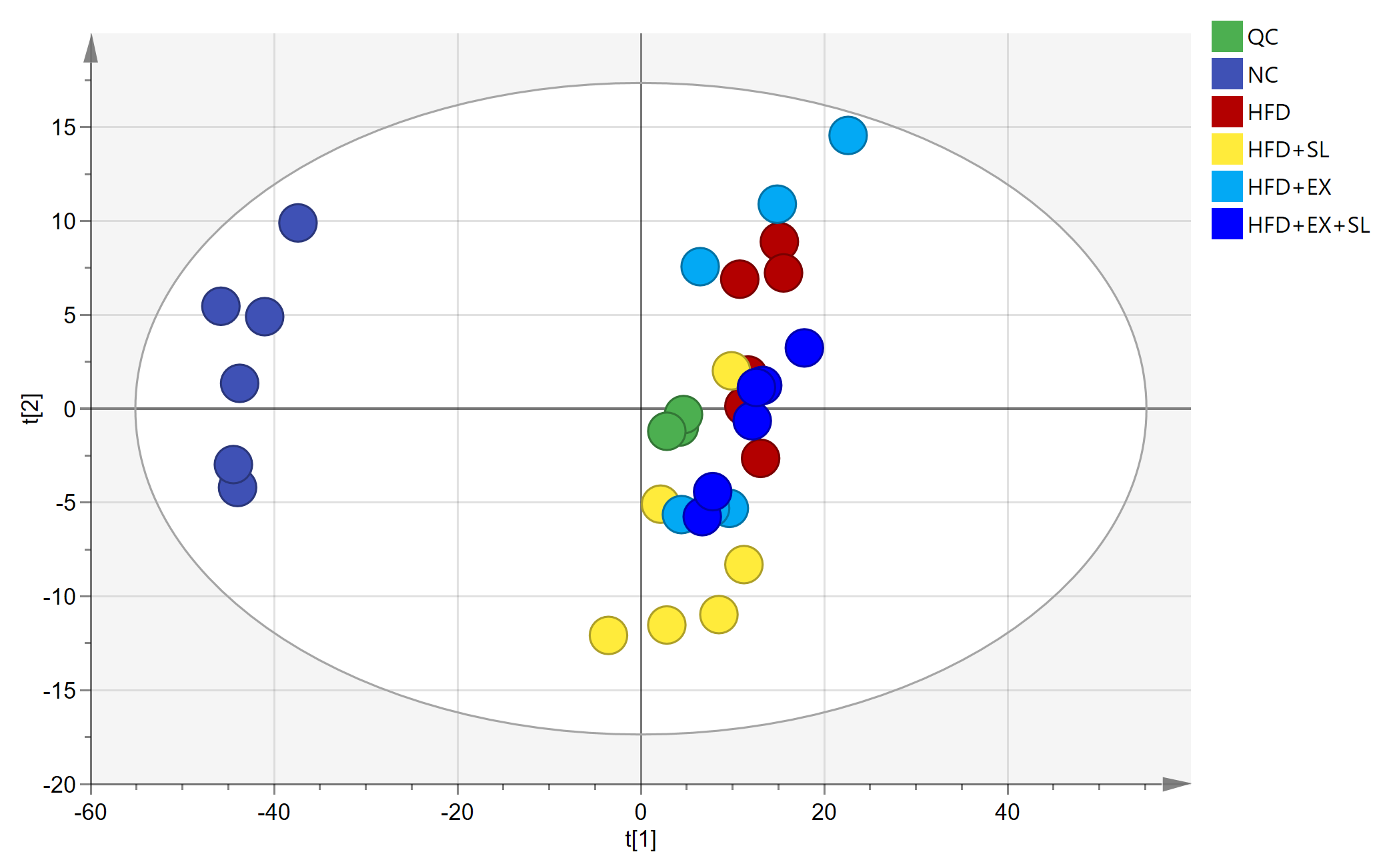

Supplement: Supplementary file 1 [file molecules-24-03943-s001.zip › Figure S3.tif]

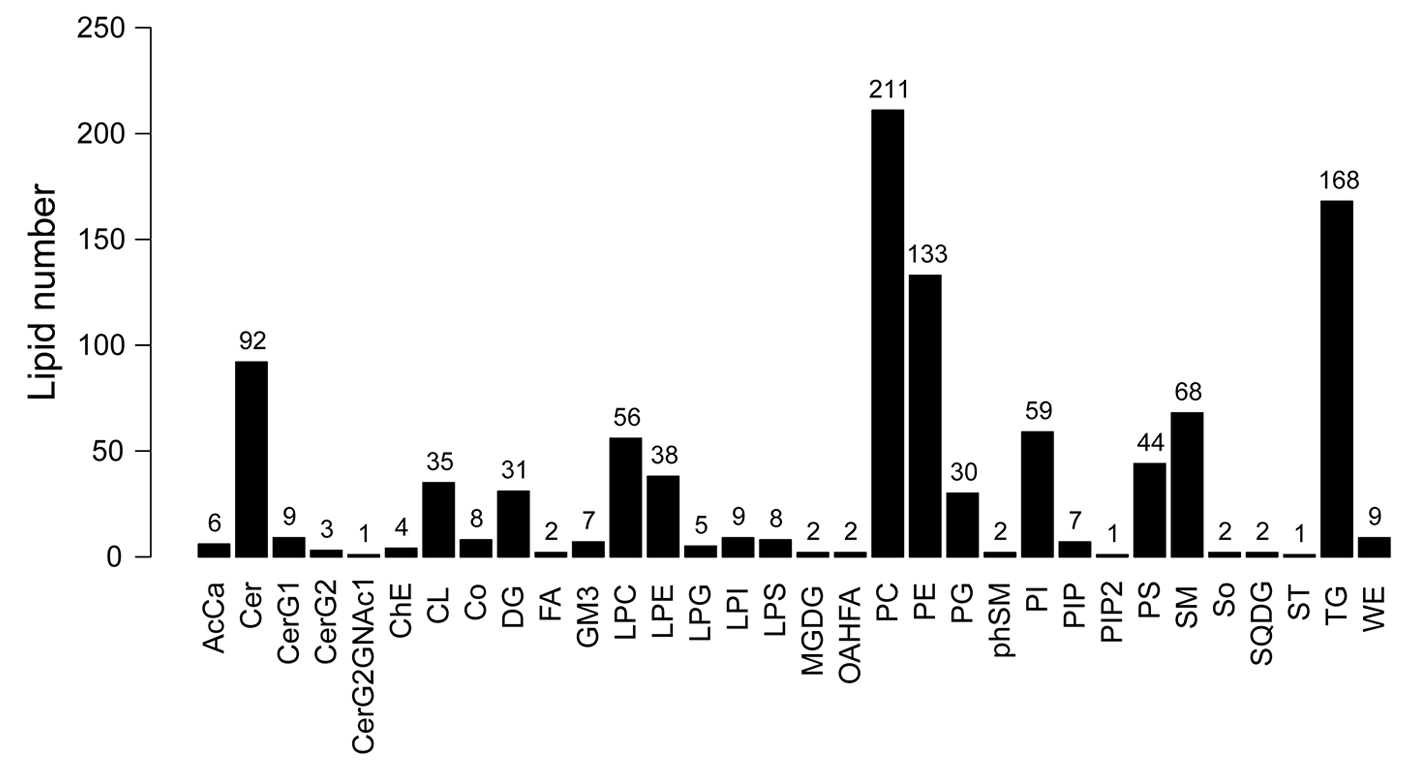

Supplement: Supplementary file 1 [file molecules-24-03943-s001.zip › Figure S4.tif]

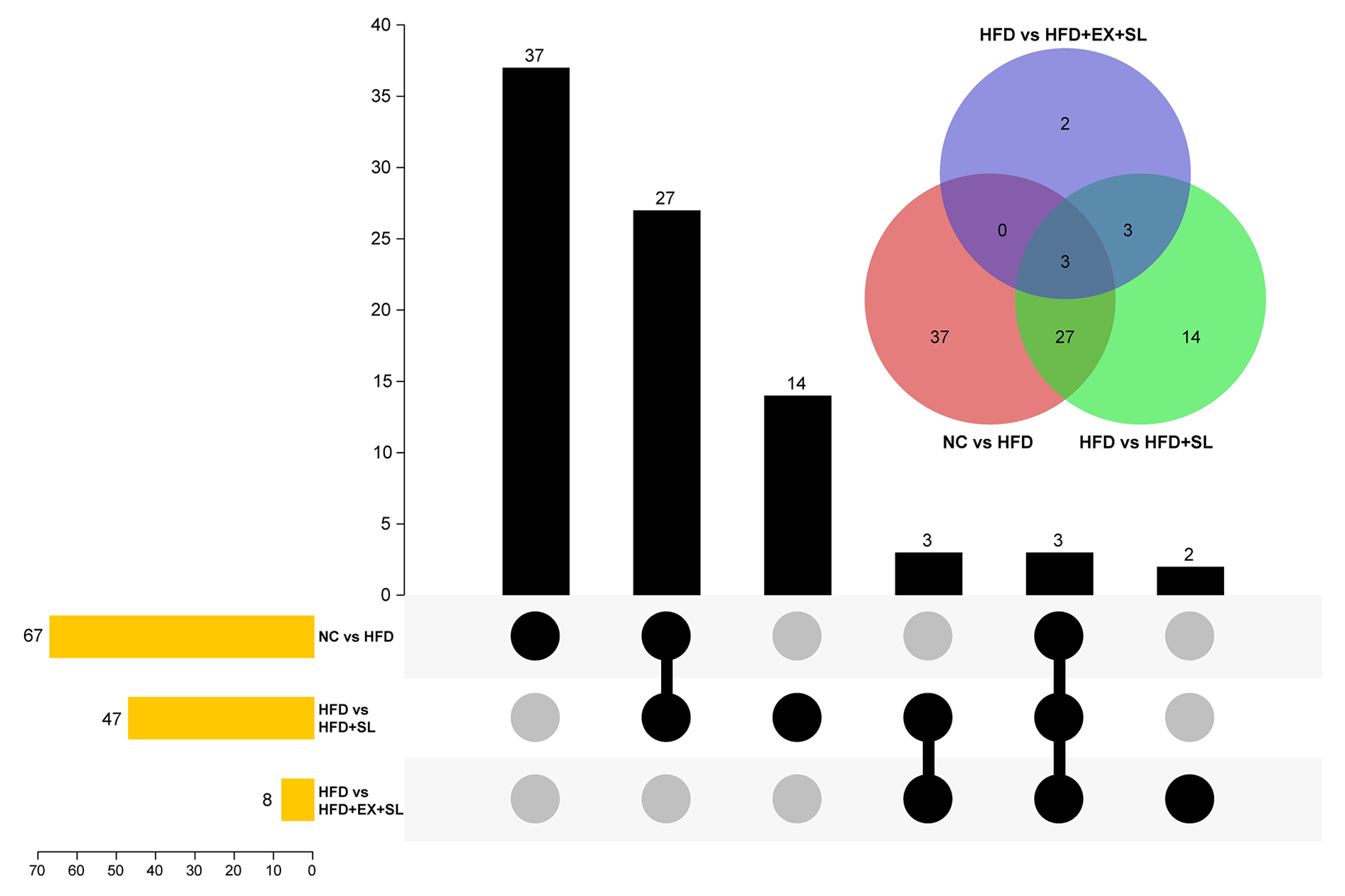

Supplement: Supplementary file 1 [file molecules-24-03943-s001.zip › Figure S5.tif]

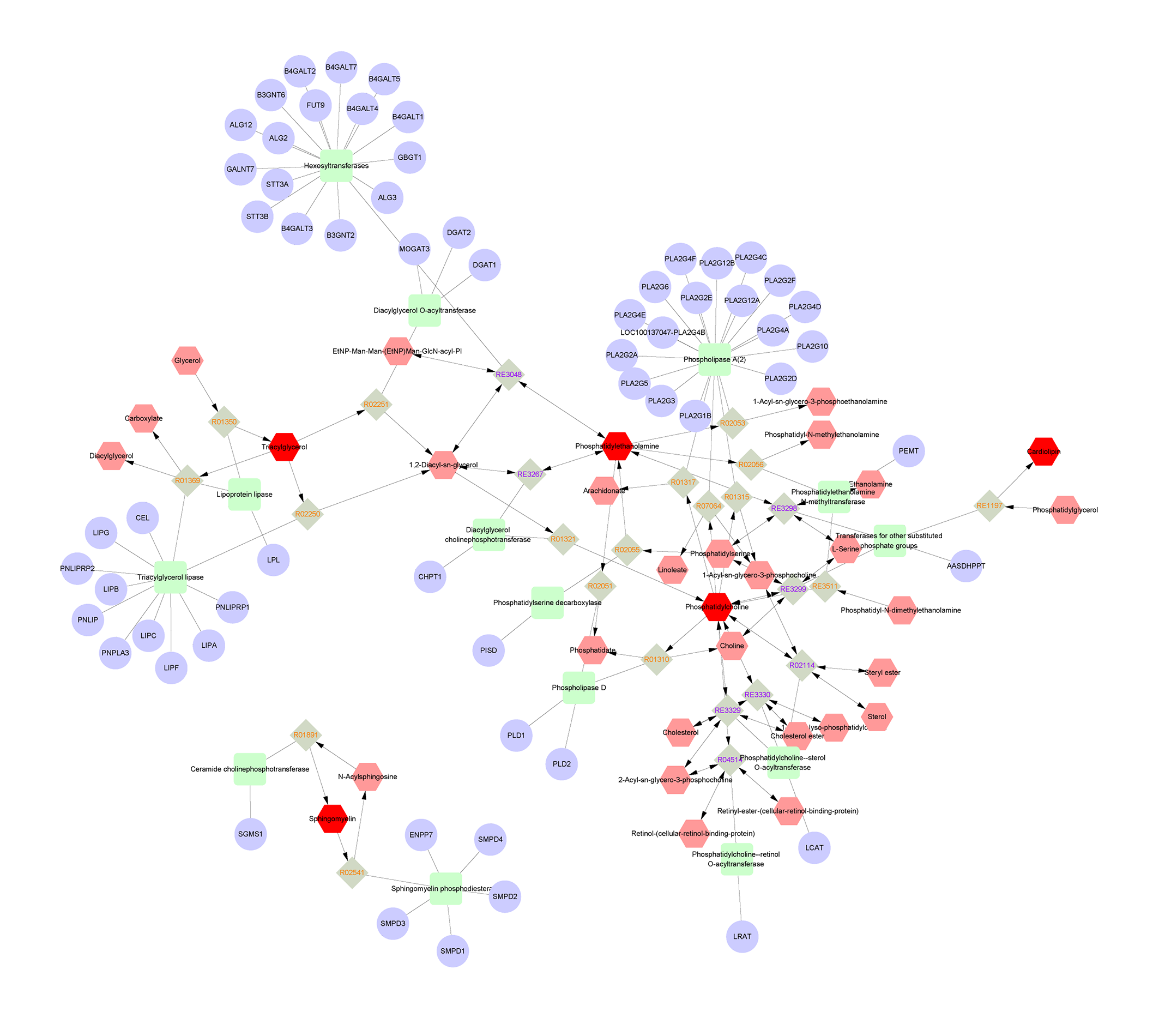

Supplement: Supplementary file 1 [file molecules-24-03943-s001.zip › Figure S6.tif]
